# Alliance Ruptures and Resolutions in Personality Disorders

**DOI:** 10.1007/s11920-020-01212-w

**Published:** 2020-12-11

**Authors:** Nathalie Schenk, Lukas Fürer, Ronan Zimmermann, Martin Steppan, Klaus Schmeck

**Affiliations:** 1 Child and Adolescent Psychiatric Research Department, Psychiatric University Hospitals of the University of Basel, Basel, Switzerland; 2 Division of Clinical Psychology and Psychotherapy, Faculty of Psychology, University of Basel, Basel, Switzerland

**Keywords:** Rupture resolution, Personality disorder, Alliance

## Abstract

**Purpose of Review:**

This review provides an overview of the state of research on alliance ruptures and resolutions in the treatment of personality disorders (PDs). We discuss frequently used instruments to measure alliance ruptures and resolutions. We discuss the effectiveness of rupture resolution processes and highlight possible avenues for research to explore. Innovative assessments with the potential to reveal the link of ruptures and resolutions and mechanisms of psychotherapeutic change are discussed.

**Recent Findings:**

The assessment of alliance rupture and resolutions is heterogeneous. Instruments vary largely with respect to a direct or indirect assessment, the time resolution of assessment (integral therapy, phase, session, event), session sampling strategy and perspectives (patient, therapist, observer). The heterogeneity in the instruments and study designs impedes comparability and interpretation of the findings. Results support the hypothesis that ruptures are more frequent in PD. Results also point towards beneficial effects of rupture resolution patterns, early alliance quality, and resolution complexity. Few studies control findings for pretreatment factors.

**Summary:**

Evidence points to the direction that rupture resolution processes can be considered a general principle of change in the treatment of PD. The concept of alliance ruptures and resolutions provides a useful tool for the management of the therapeutic alliance and its moments of deteriorations throughout the treatment course. Dimensional pretreatment personality functioning is considered a key variable in future studies to highlight what works for whom.

## Introduction

It is argued that relationship factors are not only a byproduct of the psychotherapeutic encounter but also a vehicle of change per se (Task Force Division 29: [1, 2]). Building upon a relational approach [3••], alliance research concerned with personality disorders (PDs) investigates the assumption that the process of rupture resolution brings about change [4, 5]. The NICE guidelines recommend the building and management of the therapeutic alliance as a general principle of change in the treatment of PD [6]. However, patients with PD have general difficulties in forming interpersonal relationships, including the therapeutic relationship, due to their impairments in self and interpersonal functioning [7].

Safran and Muran [8] conceptualize the therapeutic alliance as a dynamic and relational entity that is continuously built and negotiated by patient and therapist. In this negotiation process, alliance ruptures with minor or major significance emerge inevitably. Alliance ruptures are described as momentary deteriorations in the therapeutic alliance resulting from a lack of collaboration regarding the goals and tasks of the treatment or resulting from strains in the emotional bond [9•]. Withdrawal and confrontation ruptures can be differentiated by their movement dynamics (against therapist or therapy work versus avoiding therapist or therapy work). The rupture resolution is the process of recognizing, exploring, and repairing alliance ruptures with the aim to reestablish the collaboration and to explore the patient’s underlying needs and intrapsychic conflicts. The resolution process that is initiated by the therapist has the potential to enable a corrective experience that is held to be therapeutic in itself [4]. Safran and Muran [5] suggest that alliance ruptures disclose a window into core interpersonal schemes. Insofar, the therapeutic relationship enables an interpersonal learning field in which representations of the self and others, interpersonal schemes, and the needs for agency versus communion can be probed and adapted [3••]. Resolution strategies can be immediate, focusing on the reestablishment of the collaboration, or expressive, exploring the patients’ needs and intrapsychic conflicts underlying the alliance rupture [10].

Schenk et al. [11•] provide a detailed description of the occurrences of ruptures and resolutions in therapies with adolescents with borderline personality disorder (BPD). The most typical alliance ruptures were minimal response, denial, avoidant storytelling, patient defends self against therapist, complaints against therapist, and rejecting therapist intervention. Therapists typically used the resolution strategies to invite the patient to discuss thoughts and feelings about the therapist or some aspect of therapy, to validate the patient’s defensive posture and to illustrate tasks or provide a rationale for treatment (Schenk et al. [11•]). The following transcript demonstrates a short rupture-resolution sequence of a therapy session with a 16-year-old adolescent with BPD:

- T: How are you?
- P: Not good.
- T: Not good?
- P: No. (Pause) => Minimal response rupture
- T: what happened?
- P: I do not know. (Pause) => Minimal response rupture
- T: Is anything bothering you?
- P: No. => Denial Rupture
- T: How does it come you feel not good?
- P: I do not know, it’s like always. => Minimal response rupture
- T: And what does it mean when you say that you are not doing well, any thoughts or feelings about that? => Resolution “therapist invites the patient to discuss thoughts or feelings”
- P: I do not feel happy, just empty.
- T: Mmmh. Do you already know that feeling from yourself?
- P: I do not know. (Long pause) => Minimal response rupture
- T: Hmm, I have the feeling we are stuck here right now. I have the feeling you could feel pressured by me asking you all these questions. Do you feel this way with me?
- Resolution attempt “the therapist discloses his/her internal experience of the patient-therapist interaction”.

## Instruments of Alliance Ruptures and Resolutions

Alliance ruptures and resolutions can be measured with direct or indirect instruments, through different perspectives (patient, therapist observer), on different assessment levels (session, specific time windows, episodes, markers, speaking turns, utterances) and at different time points (successive therapy sessions, phases, specific sessions, between sessions, retrospective in interviews).

Table 1 presents frequently used direct and indirect instruments of alliance ruptures. The *direct* assessment is based on observer ratings or retrospective self-report questionnaires aiming directly at rupture resolution episodes. The *indirect* assessment means that the alliance is measured and inferences towards alliance ruptures and resolutions are drawn from sudden and significant fluctuations in the global alliance. Eubanks-Carter et al. [24] provide an overview of the quantitative naturalistic methods for detecting alliance ruptures on the basis of global alliance measures.

**Table 1:** Direct and indirect instruments of alliance ruptures

	Instruments	Perspective	Details
Direct instruments	Rupture Resolution Rating System (3RS; [12])	Observer rating	Contains 7 withdrawal and 7 confrontation rupture markers as well as 10 resolution markers. Markers are rated on a significance rating scale and an overall resolution rating scale. Units of coding are speaking turns, 1- or 5-min windows or defining start and stop markers for each episode.
	Collaborative Interaction Scale- Revised (CIS-R; [13])	Observer rating	29-item rating scale with two subscales CIS-P and CIS-T. CIS-P defines direct and indirect rupture markers and direct and indirect collaborative processes of the patient. CIS-T defines direct and indirect collaborative interventions, rupture and therapist interventions. Coding is done within narrative units. Ruptures and resolutions can be coded for both the patient and the therapist.
	Harper’s [14, 15] manual of rupture markers	Observer rating	Defines 10 markers of confrontation ruptures and 8 markers of withdrawal ruptures. The analysis is performed on the level of speaking turns.
	The Structural Analysis of Social Behavior (SASB; [16])	Observer rating	Measures interpersonal behavior of the patient and therapist with the octants “focus on other” and “focus on self” with two orthogonal dimensions interdependence (from autonomy to involvement) and affiliation (from hostility to friendliness). Alliance ruptures were rated as patient behaviors of appeasing, avoiding and blaming. Resolutions were rated as patient and therapist expressing and therapist affirming and directing [9•].
Direct and indirect instruments	Post-Session- Questionnaire [17]	Patient, therapist	Combines the session evaluation questionnaire (SEQ; [18]) and *direct* questions of whether or not there was a problematic event in the relationship during the session and when these events occurred. Patients rate the tension of the problematic event on a 5-point Likert scale. The SEQ assesses the session impact (subscales depth and smoothness) and post-session feelings of positivity and arousal.
Indirect instruments	Working Alliance Inventory Short Form (WAI-SF; [19])	Patient, therapist, observer	12-item self-report with three subscales goals, tasks and bond of the therapeutic alliance. It is mostly administered at the end of sessions.
	California Psychotherapy Alliance Scales (CALPAS; [20])	Patient, therapist, observer	The subscales patient working capacity, patient commitment, working strategy consensus and therapist understanding and involvement are assessed. Observer rating is done on the session level.
	Vanderbilt Therapeutic Alliance Scale-Revised Short Form (VTAS-R SF; [21])	Observer	5-item rating scale with two subscales patient contribution and patient-therapist interaction. The items reflect Bordin’s theoretical constructs bond, goals and tasks. Items are scored on a 6-point Likert scale. The coding is done on session level.
	Alliance Negotiation Scale (ANS; [22])	Patient	12-item self-report scale that assesses the degree of constructive negotiation of disagreements about tasks and goals from the perspective of the patient.
	The Agnew Relationship Measure (ARM; [23])	Patient, therapist	26-item questionnaire measuring the quality of the therapeutic relationship from the perspective of the therapist and the patient.

The *direct* instruments with observer ratings (e.g., 3RS, CIS) enable a detailed investigation of the process of the alliance negotiation based on the event paradigm [25]. This fine-grained analysis is particularly of interest to understand underlying principles of change through rupture resolution processes. Using task analysis, the performance of rupture resolutions can be studied in detail as done by Aspland et al. [26], Bennett et al. [27•], and Daly et al. [28]. Furthermore, with descriptive studies the incidence and phenomenological quality of ruptures and resolutions can be examined in different patient samples [11•, 29, 30]. Limitations are that the coding is time and cost intensive and therefore often restricted to small sample sizes which impedes generalizability and inferences to the treatment outcome. The *indirect* instruments derived from self-report ratings of the alliance assess changes in the quality of the alliance on a global level. They estimate the impact of possible but not directly measured ruptures and likely measure major but not minor ruptures. Indirect measures allow the investigation of large patient samples on a macro level of investigation. They are feasible for the study of within-patient and between-patient effects of rupture resolution processes in relation to the treatment outcome. However, the indirect measures are less accurate and might be biased regarding construct validity. In comparison to observer ratings, self-report measures underestimate the amount of alliance ruptures [29, 31].

## Influence of PD Traits on Rupture Resolution Processes

Theoretical and clinical considerations about the interpersonal nature of different PDs allow assumptions towards the quality of ruptures encountered with these patients [3••, 32•]. For example, withdrawal ruptures may be more frequent in patients who are overly compliant, fearful, and averse to interpersonal conflicts such as cluster C patients. Confrontation ruptures may be more frequent in patients with cluster A or B PD as they use more direct and overt strategies to disclose strains in the alliance like criticize or pressure the therapist. Unfortunately, there is no empirical evidence emphasizing these hypotheses.

Zilcha-Mano [33••] proposed to differentiate between trait-like (i.e., pretreatment tendencies of the patient to form relationships with others) and state-like components of alliance (i.e., changes in relationship functioning through the interaction with the therapist). It is hypothesized that patients with PD present with lower trait-like pretreatment interpersonal functioning that leads to problems in building and maintaining a stable working alliance. This could lead to more ruptures during the process and a lower (early) alliance quality compared to other clinical groups.

In the validation study of the 3RS, Eubanks et al. [12] found ruptures in almost every session of a psychotherapy with patients with varying diagnoses. However, Schenk et al. [11•] used the 3RS to analyze the complete treatment of a sample of ten adolescents with BPD. In 72% of the sessions, at least one rupture was observed.

Colli et al. [13] compared 15 PD patients (cluster B and C) with 15 patients suffering from other psychiatric disorders. In PD patients, rupture markers were observed more frequently than in non-PD patients (tasks and goals marker: *F* = 4.7, *p* = .031, discouragement marker: *F* = 4.0, *p* = .046). Interestingly, they also found that therapists of PD patients tended to be more hostile and less clear and used more perseverations than non-PD therapists. However, the PD therapists also provided more supportive, explicative, and expressive interventions targeting alliance ruptures than the non-PD therapists.

Tufekcioglu et al. [34] tested whether cluster C PD or personality traits impact the early alliance compared to non-PD patients. They found that PD patients rated a higher rupture intensity than non-PD patients (*F* = 16.6, *r* = .43, *p* < .01). Furthermore, they found that the pretreatment personality traits of high impulsivity, dysregulation, and lability (but not diagnoses) were associated with higher patient-reported rupture intensity. Coutinho et al. [29] found that patients with PD experience a lower alliance quality (measured with the WAI) and more frequent withdrawal and confrontation ruptures (3RS) compared to patients with depression and anxiety disorders.

In conclusion, there is evidence that confrontation and withdrawal ruptures are more frequent in PD than other clinical samples. Differences between PD and non-PD patients seem to also surface when observing therapist behavior. The findings can be interpreted in the light of a trait-like alliance component of lower interpersonal functioning in PD patients, meaning that the quality of the therapeutic relationship is endangered from the beginning on by the severe interpersonal problems that are at the core of PD symptomatology.

## The Effects of Ruptures and Resolutions

Studies concerned with the correlation between alliance and outcome focus on different characteristics of the alliance, depending on the instruments used. We review the outcomes of meta-analyses, literature investigating early alliance, rupture resolution processes (e.g., significant fluctuations in WAI) and literature focusing on resolutions and their complexity. When trying to unveil the state-like components of alliance leading to change (curative interactions with therapists), one has to control for trait-like components (e.g., pretreatment personality functioning), setting the preconditions for a beneficial interpersonal relationship. Not all studies reviewed here do that.

Up to date there are two meta-analyses that report moderate effects of rupture resolution processes on the treatment outcome [9•, 35]. Safran et al. [35] summarized studies using indirect instruments of alliance ruptures. In this meta-analysis, the number of rupture resolution episodes was positively related to treatment outcome (*r* = .24, 95% *CI* [.09, .39], *p* = .002, *k* = 3, *n* = 148). The meta-analysis of Eubanks et al. [9•] confirmed that rupture resolution processes are positively associated with the treatment outcome when using direct as well indirect measures (*r =* .29, *CI* [.10, .47], *d =* .62, *p* = .003, *k* = 11, *n* = 1314). The effect was independent of type of treatment or type of instruments used. However, the timing of the assessment impacted the rupture resolution outcome relationship. An assessment in early sessions resulted in a lower association (*r* = .13, *z* = 1.90, *p* = .06), whereas an assessment across the complete course resulted in a stronger rupture resolution outcome association (*r* = .38, *z* = 3.13, *p* = .002). This is an encouraging result when hypothesizing that late sessions may better reflect state-like alliance components, whereas early sessions could be more influenced by the trait-like pretreatment conditions.

Considering early alliance, Strauss et al. [36] found that patients (obsessive-compulsive PD and avoidant PD) who showed higher pretreatment personality pathology presented with a lower early alliance (*r* = − .40, *p* < .05), and in turn, better early alliance predicted a higher number of sessions attended (*r* = .38, *p* < .05). Also, better early alliance predicted gains in all post-treatment outcome measures (personality pathology: *r* = − .40, *p* < .05, depression score: *r* = − .49, *p* < .01). Also, Muran et al. [37] found that lower early rupture intensity was associated with higher post-treatment interpersonal functioning (cluster C and NOS, patient rated: *r* = − .35, *p* < .01; therapist rated: *r* = − .32, *p* < .01). In a youth BPD sample, Gersh et al. [38] found that more early ruptures were associated with poorer outcomes in social functioning (*r* = .32, *p* < .05).

There is evidence for rupture resolution patterns (significant fluctuations or dips in alliance implying ruptures, returning to baseline and above, implying resolution). Schenk et al. [11•] assessed ruptures with the 3RS on a session-by-session basis. Nonlinear rupture trajectories were found on the individual level with high intra- and interindividual differences. In adolescent patients with BPD, ruptures tended to emerge in phases. Also Stevens et al. [39] found that 50% of patients (cluster C and NOS) showed local rupture resolution patterns. There was however no association to be found between the occurrence of rupture resolution patterns and outcome. On the other hand, Strauss et al. [36] found that 56% of their sample showed rupture resolution patterns. Higher pretreatment personality pathology (cluster C) resulted in less rupture resolution patterns (*r* = .43, *p* < .05). Occurrence of rupture resolution patterns in turn predicted improvement in personality pathology (*r* = − .53, *p* < .01) and depression (*r* = .41, *p* < .05).

When looking at resolutions, Muran et al. [37] found that patients (cluster C and NOS) who experience more resolutions of ruptures were less likely to drop out (*r* = − .29, *p* < .05). In contrast, for adolescents with BPD, Schenk et al. [11•, 40] showed that although dropouts experienced a higher frequency of alliance ruptures per session in comparison to the completers, therapists of the dropout patients applied an equal proportion of resolutions to ruptures as the therapists of the completers. This indicates that the mere number of resolutions was not protective of dropping out in this particular sample. In the same vein, Gersh et al. [38] found that a higher number of resolutions in late sessions were associated with improvement in BPD symptoms (*r* = − .67, *p* < .05). Daly et al. [28] demonstrated that the complexity of resolution strategies was associated with the treatment outcome in a sample of three recovered and three unrecovered adolescent patients with BPD. Complexity was conceptualized by the number of stages of Bennet’s [27•] resolution stage model employed by therapists. The resolution stage model was defined in a task analytical effort and engulfs nine hierarchical (sequential) stages, namely acknowledgment, exploration, linking and explanation, negotiation, consensus, understanding and assimilating warded off feelings, further explanation, change to patterns/aim, and closure. Daly et al. [28] showed that resolved rupture episodes are characterized by more and higher (negotiation to closure) model stages. Further, therapists of recovered patients used more and higher stage resolutions than therapists of unrecovered patients who more often remained at a lower stage of resolution. In contrast, Boritz et al. [30] found that withdrawal ruptures tended to persist over time in the unrecovered but not the recovered patients (BPD) despite the degree to which they were resolved in the prior session.

In summary, there is first meta-analytical evidence for the beneficial effect of rupture resolution processes during psychotherapy. When looking at PD literature, early alliance seems to influence dropout status and positively predict outcome. However, Strauss et al. [36] found that early alliance is associated with pretreatment pathology, indicating that the beneficial effects of early alliance are found in patients that are able to build a stable therapeutic relationship in the early phase of therapy. In the same vein, the underlying principles of change in rupture resolution patterns still remain unclear given their possible link to pretreatment personality pathology. The studies concerned with resolutions and their complexity during the process point towards favorable effects of higher order resolutions and resolutions in the later phase of therapy. However, they also indicate that in some cases the amount and the complexity of resolutions remain futile. The contextual factors (therapist effects, patient interpersonal dysfunction) hindering the therapeutic effect of offered resolutions remain an important avenue for future research.

## Rupture Resolution Training

Eubanks-Carter et al. [41] have developed an alliance-focused training (AFT). Safran and Muran’s publication on *Negotiating the Therapeutic Alliance: A Relational Treatment Guide* [8] serves as a training manual. Muran et al. [42] have tested the additive effect of AFT during a 30-session CBT protocol for cluster C PD on interpersonal behavior (SASB). Therapists were introduced to AFT at different time intervals (after session 8 or 16) controlling for patient, therapist, and patient-therapist interactional effects. For patients, the introduction of AFT predicted a decrease in following behavior, an increase in expressiveness and a trend was found towards less avoidance and appeasement. For therapists, the onset of AFT training predicted a decrease in therapist blaming and directing and an increase in therapist affirmation and expressiveness. Findings demonstrate that the expressing behavior of the patient was positively related to treatment outcome and patient appeasing and blaming were negatively related to treatment outcome. The authors insofar were able to show that AFT resulted in beneficial changes of interpersonal processes in a CBT setting.

Although the study of Muran et al. [42] demonstrates meaningful changes in interpersonal behavior, the impact of rupture resolution training on the outcome level remains sparse. The meta-analyses of Eubanks et al. [9•] did not support a significant effect of a rupture resolution training on the treatment outcome.

## Need for New Methods in Future Research

The finding that a better alliance is associated with a better treatment outcome has been confirmed in numerous studies [43–45]. However, in these study designs, the alliance is treated as a fixed effect factor that is measured most often at the beginning of the therapy. Therefore, the dynamic quality of the alliance is neglected. With advanced statistical methods and study designs, the state- and trait-like components in the alliance-outcome association can be distinguished. This allows to analyze to what extent the alliance acts as a precondition to the therapeutic process (trait-like general relationship tendencies of the patient) or as a dynamic entity of the therapeutic relationship (i.e., state-like like rupture resolution processes) that operates as a vehicle of change [33••]. However, the video analysis of critical events is very labor- and cost-intensive. This results in studies with either low or no repeated measurement or in studies with small sample sizes. Future research should therefore invest in automated methods to describe alliance characteristics using physiological [46, 47], vocal [48, 49], facial [50], and movement [51] information streams. Interpersonal processes have been proposed to mirror the quality of interactions; especially synchrony has been proposed as an integrative framework for the therapeutic alliance [52•]. This theoretical framework allows to focus on objectively measurable interpersonal behavior and to complement the subjective experience and linguistic interaction studied so far in alliance research [53]. Alternatively, machine-learning could be used to approximate critical events in psychotherapy based on automated information streams and to mark them for further manual evaluation. For example, minimal response withdrawal ruptures could be approximated by automated silence detection [40] and speaker diarization [54]. The extent of nonverbal movement synchrony has been associated with patient-rated relationship quality [51], dropout status, and improvement [55]. Reich et al. [49] found that vocal synchrony in turn was negatively associated with the patient-rated working alliance. The here proposed methods assess alliance ruptures only indirectly but on a fine-grained temporal level in large samples sizes over the whole psychotherapeutic process. This would allow to study bridging concepts like synchrony or emotion regulation in relation to working alliance processes for PD specifically.

## Conclusion

Alliance ruptures and their resolution in PD are studied through direct (observer-based methods: 3RS, CIS) and indirect methods (e.g., questionnaires: PSQ, WAI). Horvath [56] estimates over 70 different instruments that operationalize the alliance based on different theoretical constructs. The biggest problems of the field are objectivity and comparability. Studies are sparse and highly heterogeneous (PD cluster, research aims, session sampling, methods), impeding the comparability and interpretation of findings. Concordance of direct and indirect measures of alliance ruptures must be considered low [31, 57]. In order to avoid a forest of unconnected and therefore hard to summarize findings, methodological objectivity should be considered a key interest for the field.

Throughout the literature there is frequent use of cluster C patient samples; cluster A and B studies are highly encouraged. It is hypothesized that PD diagnoses influence the quality of ruptures encountered through their characteristic interpersonal constraints [3••, 32•]. It is therefore important to acquire and compare findings for all different types of PD. Moving towards the dimensional ICD-11 diagnosis, future studies should include dimensional personality functioning as covariate variables to embed findings. Results by Strauss et al. [36] or Muran et al. [37] already point towards the direction that personality functioning is a more feasible moderator and outcome variable than the number of SCID items. Further, few studies test for trait-like pretreatment factors moderating the alliance outcome association. Dimensional pretreatment personality functioning (DSM-5; [58•, 59•]) is considered an important variable for future studies, in order to report what has worked for whom.
